# Synchronous primary HPV-associated squamous cell carcinomas of the cervix and fallopian tube: a case report

**DOI:** 10.3389/fonc.2026.1776043

**Published:** 2026-06-03

**Authors:** Juan Lu, Changqian Chen, Yanfang Bai, Min Kang

**Affiliations:** 1 Department of Gynecology, Zhuhai Hospital of Integrated Traditional Chinese and Western Medicine, Zhuhai, China; 2 Department of Pathology, Zhuhai Hospital of Integrated Traditional Chinese and Western Medicine, Zhuhai, China

**Keywords:** cervical cancer, human papillomavirus infection, multiple primary malignant neoplasms, primary fallopian tube carcinoma (PFTC), squamous cell carcinoma

## Abstract

Cervical squamous cell carcinoma has been proven to be associated with human papillomavirus(HPV) infection, while the etiology of fallopian tube squamous cell carcinoma remains unclear and there are few literature reports. Clinically, concurrent cases of the two malignancies are even more rare. A female patient presented to our hospital with irregular vaginal bleeding. She underwent hysteroscopic surgery, and the postoperative pathology indicated cervical cancer. She subsequently received laparoscopic surgery, and postoperative pathology unexpectedly confirmed squamous cell carcinoma of the fallopian tube. We speculate that this represents a case of HPV-associated double primary squamous cell carcinomas. At present, the patient’s general condition is stable after systematic treatment. This case reminds us to further understand the relationship between HPV infection and gynecological malignancies, as well as the benefits of rational application of opportunistic salpingectomy (OS), and we should strive for early diagnosis and treatment of tumors.

## 1 Introduction

Among malignant tumors of the female reproductive system, reports of concurrent cervical cancer and fallopian tube cancer are exceedingly rare. This article reports a case of HPV-associated double primary squamous cell carcinoma. It is hypothesized that the concurrent development of fallopian tube and cervical cancers may be related to HPV infection. The aim is to draw clinical attention to HPV infection, enable early prevention and treatment, and provide support for subsequent research on the pathogenesis of concurrent cervical and fallopian tube carcinoma.

## 2 Case presentation

A 45-year-old women presented to our inpatient department on August 27, 2023, with irregular vaginal bleeding lasting over 20 days. This patient has had regular menstrual cycles in the past. She was G3P1 with two induced abortions. Her past medical history includes laparoscopic excision of a left ovarian cyst over ten years ago, although details are unclear. In 2020, she underwent a cervical conization for high-grade squamous intraepithelial lesion (HSIL).And during follow-up, she had intermittent ThinPrep cytology test(TCT) and HPV test, which showed no abnormalities. She has no family history of malignancy. She underwent systematic examinations, the transvaginal ultrasound revealed heterogeneous echotexture of the myometrium and endometrium (thickness in 1.1 cm), with areas of decreased echogenicity interspersed and patchy hyperechoic regions, and the largest area is approximately 1.0×0.5 cm with indistinct borders. Serum tumor markers CA125,CA153 and CA19–9 were all within normal limits. High-risk HPV DNA testing detected HPV type 16 was positive (+). TCT revealed no intraepithelial lesions or malignancy(NILM). Colposcopic examination confirmed a type II transformation zone on the cervix, with thin acetowhite epithelium and early regression. On August 29th, the patient underwent hysteroscopic resection of endometrial polyps and endocervical curettage. The postoperative pathology revealed endometrial polyps within the proliferative phase endometrium and occasional cervical canal epithelium exhibiting a HSIL. The cervical canal epithelium showed evidence of HSIL. Immunohistochemical (IHC) staining results indicated the following, P16 was positive in the squamous epithelium, and the Ki-67 hotspot proliferation index was approximately 90%. The patient was scheduled for cervical conization to rule out malignancy but requested a total hysterectomy instead. After being informed of the potential outcomes and risks, she underwent a total laparoscopic hysterectomy (TLH) with bilateral salpingo-oophorectomy(BSO) on September 4. Postoperative pathology revealed the following:(1) (Cervical canal) HPV-associated non-keratinizing squamous cell carcinoma, infiltrating the superficial cervical muscle layer (with an approximate depth of invasion of 1 mm), adenomyosis, and proliferative phase endometrium.(2) (Left fallopian tube) HPV-associated non-keratinizing squamous cell carcinoma, infiltrating the tube’s lamina propria, accompanied by a HSIL and a mesenteric paramesonephric duct cyst.(3) (Right fallopian tube) HSIL with a mesenteric paramesonephric duct cyst. IHC staining results indicated the following: tumor cell markers on Slide of the left fallopian tube, CEA and CK7 were positive, MLH1, MSH2, MSH6 and PMS2 were positive, Napsin A was negative, P53 was mutant, PAX-8 was negative, WT1 was negative, P16 and P40 were intense positive. On slide of the cervical canal, P53 was mutant, PAX-8 was negative, WT1 was negative, ER was partially positive, PR was negative, P16 was positive, and Ki-67 hotspot proliferation index was approximately 90%. The anatomopathologists from the Second Affiliated Hospital of Guangzhou Medical University carefully reviewed the slides. The patient underwent contrast-enhanced full abdominal MRI which revealed no positive space-occupying lesions and swollen lymph nodes. A supplementary surgery was performed on September 18. Postoperative pathology revealed no evidence of cancer metastasis in any examined lymph nodes, including pre-sacral (0/1), left common iliac (0/3), right common iliac (0/5), left internal iliac (0/5), right internal iliac (0/3), left external iliac (0/3), right external iliac (0/6), left obturator (0/2), right obturator (0/4), left para-aortic (0/3) and right para-aortic (0/2). No cancer involvement was observed in the tissue specimens submitted for examination, including bilateral ovaries with corresponding pelvic infundibular ligaments and round ligaments, paracervical and vaginal tissue, and greater omentum. The patient was diagnosed with stage IA1 cervical squamous cell carcinoma and stage IA fallopian tube squamous cell carcinoma. Regarding treatment, the patient refused genetic testing and subsequently underwent adjuvant chemotherapy with three cycles of paclitaxel and carboplati at a local hospital. Through telephone consultation, the patient underwent standard follow-up, and the most recent examination in October 2025 showed that her condition was in complete remission.

## 3 Discussion

Multiple primary malignant neoplasms (MPMN) refer to the simultaneous or sequential occurrence of two or more primary malignant tumors in one or more organs of the same individual, either simultaneously or sequentially. When the onset interval is less than 6 months, it is termed synchronous multiple primary malignant neoplasms; when the interval exceeds 6 months, it is termed metachronous multiple primary malignant neoplasms (1, 2). In the pathogenesis of MPMN within the female reproductive system, the widely accepted theory is the “extended Müllerian duct system”. This theory posits that the cervix, endometrium, ovarian epithelium, and fallopian tubes all originate from the Müllerian duct system. Their responses to identical carcinogenic stimuli involves similar mechanisms, potentially leading to the simultaneous occurrence and progression of multiple primary malignant tumors (3).

**Figure 1:**
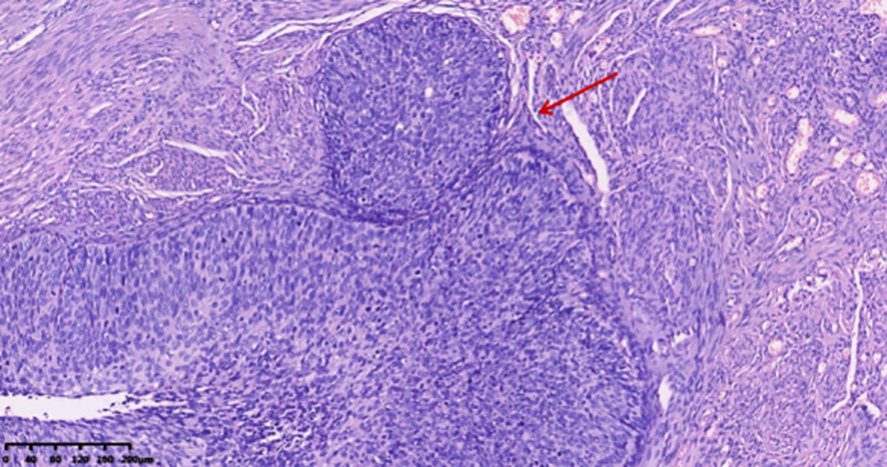
At the cervical internal os, focal superficial invasive squamous cell carcinoma was observed (HE×100).

**Figure 2:**
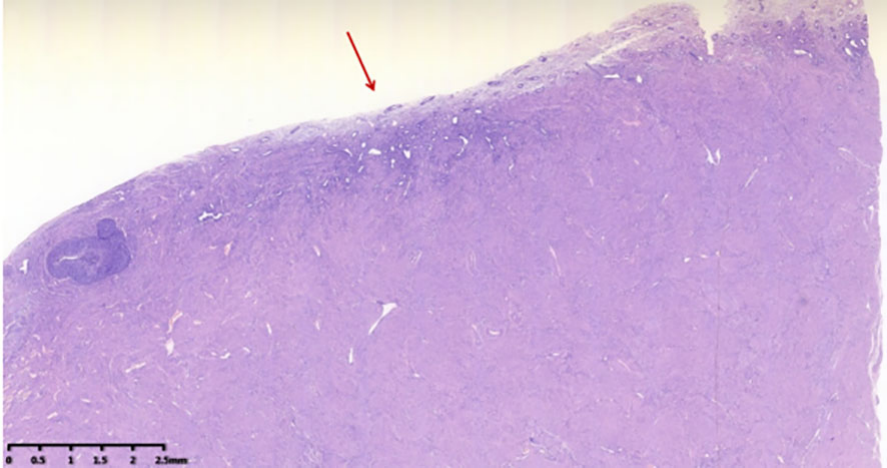
In the endometrium, proliferative endometrium was observed, with no tumor infiltration (HE×10).

**Figure 3:**
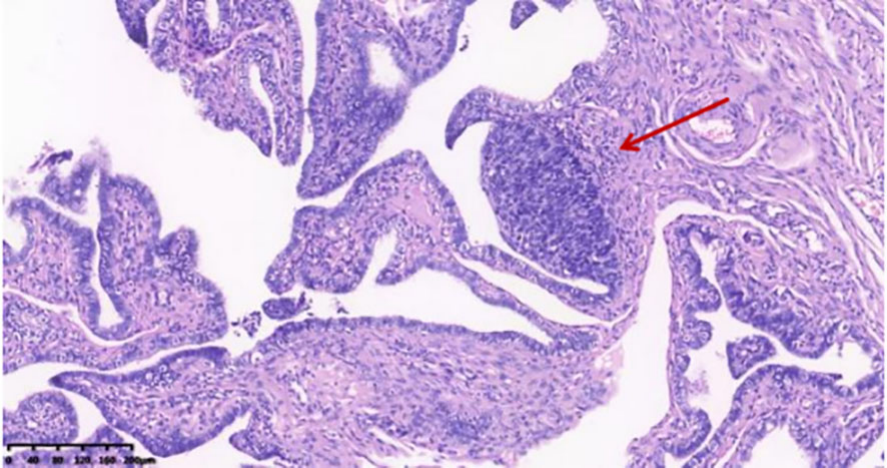
In the mucosal layer of the right fallopian tube, focal HSIL was observed (HE×100).

**Figure 4:**
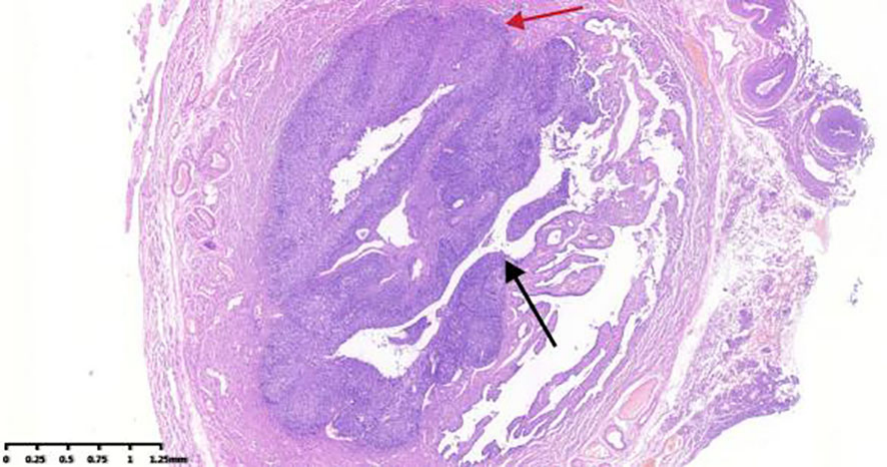
On the surface of the mucosa of the left fallopian tube, a progressive transition from normal columnar epithelium to HSIL was observed(red arrow).Squamous cell carcinoma infiltrates the underlying lamina propria and muscular layer, with an infiltration depth of approximately 2 mm. The serosal layer of the fallopian tube was not invaded (black arrow) (HE×20).

**Figure 5:**
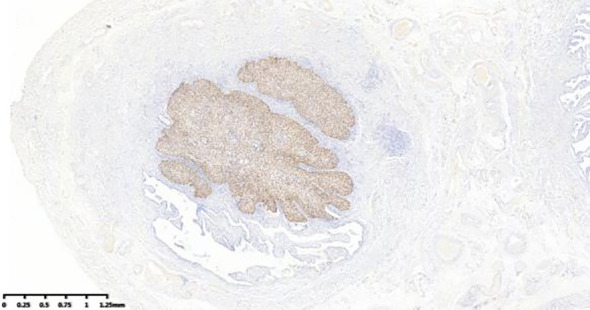
IHC result of left fallopian tube lesion: P40(+).

In this case, concurrent squamous cell carcinoma of the cervical canal and fallopian tubes are presented. It is necessary to determine whether the tumors are primary or metastatic. Given the patient’s history of high-grade cervical intraepithelial neoplasia, current confirmation of HPV16 positivity, and pathological diagnosis of cervical squamous cell carcinoma (**Figure 1**), primary cervical squamous cell carcinoma is considered. Rare cases have documented direct upward spread of cervical carcinoma to the endometrium, fallopian tubes, and ovaries, resulting in multiple squamous cell carcinomas (4). However, in this case, the endometrium exhibits proliferative changes without evidence of malignancy (**Figure 2**), suggesting non-continuous lesions in the cervix and fallopian tubes. Therefore, tubal lesions are not considered metastatic malignant tumors. The pathological diagnostic criteria for PFTC are as follows: a. the main tumor arises from the endosalpinx, with microscopic findings primarily showing involvement of the tubal mucosa presenting a papillary structure; b. the histological pattern reproduces the epithelium of tubal mucosa; c. transition from benign to malignant tubal epithelium is demonstrable; d. the ovaries or endometrium are either normal or contain a tumor that is smaller than the tumor in the tube. Meeting any one of these criterion is sufficient for a clear diagnosis (5, 6). This patient had no other history of intrauterine procedures except induced abortion. Postoperative pathology revealed HSIL in both fallopian tubes, the left fallopian tube showed a transition zone from normal epithelium to HSIL and then to squamous carcinoma (**Figure 3**, **4**), which indicated the coexistence of benign and malignant lesions in the left fallopian tube. This finding meets the diagnostic criteria for PFTC. Additionally, common histological subtypes of tubal carcinoma include serous carcinoma, endometrioid carcinoma, carcinosarcoma, clear cell carcinoma, and mucinous carcinoma, among which high-grade serous carcinoma is the most prevalent. In this case, no adenocarcinoma component was identified microscopically, and PAX-8 and WT-1, which are highly expressed in serous tumors (7, 8), were both negative (–). While the squamous carcinoma marker P40 was positive (+) (**Figure 5**), this ruled out serous tumor origin, leading to a final diagnosis of primary HPV-associated non-keratinizing squamous cell carcinoma of the fallopian tube. The dissemination and metastasis of PFTC closely resemble those of ovarian carcinoma, occurring via implanting metastasis, direct extension, lymphatic metastasis, and hematogenous metastasis. Among these, implanting metastasis is the most common route. In this case, squamous cell carcinoma was simultaneously observed in both the fallopian tube and cervix. Pathology revealed no cancer foci in the ovaries, uterine body surface, or greater omentum, ruling out implanting metastasis or direct spread. Lymph node and lymphovascular space infiltration were negative, excluding other metastatic routes. In summary, the cervical carcinoma does not correspond to metastasis from tubal carcinoma. Therefore, this patient presents with a rare dual primary squamous cell carcinoma of the reproductive tract.

PFTC originates from the epithelial lining of the fallopian tubes. This disease is extremely rare clinically, accounting for approximately 0.14% to 1.8% among all female genital malignancies (9). Its pathogenesis remains unclear, with current research linking it to various hormonal, reproductive, genetic, and environmental factors. Additional contributing factors include chronic pelvic inflammatory disease, mutations in *BRCA1*, *BRCA2*, and *HER2/Neu* genes, and infertility (10, 11).Primary squamous cell carcinoma of the fallopian tube (PSCCFT) is even more rarely reported. Squamous cell carcinoma originates from squamous epithelium or tissues undergoing squamous metaplasia. The possible origin mechanisms of PSCCFT are speculated to involve the following three aspects. First, squamous metaplasia: Long-term chronic inflammation or foreign body irritation may induce squamous metaplasia in the columnar epithelium of the fallopian tube mucosa, which may potentially undergo malignant transformation into squamous cell carcinoma. Second, the embryonic remnant theory: In 1829, Lobstein and Recamier proposed that cancer originates from embryonic remnants in adults, giving rise to the embryonic remnant hypothesis. This hypothesis suggests that the fallopian tube may contain minute clusters of embryonic squamous epithelial cells that undergo metaplasia and subsequent malignant transformation. Furthermore, the fallopian tubes and ovaries both belong to the Müllerian duct system, and considering the pathogenesis of primary ovarian squamous cell carcinoma, it is speculated that PSCCFT may arise from the malignant transformation of fallopian tube endometriosis or mature cystic teratomas of the fallopian tubes (12–16). The development of cervical squamous cell carcinoma is closely associated with high-risk HPV infection. This patient had a history of cervical HSIL three years ago, indicating a history of cervical precancerous lesions. The current examination revealed HPV16 positivity. Otherwise, P16/Ki-67 dual staining can identify the presence of HPV infection in the transformation zone. P16 has been used as a surrogate biomarker for HPV infection (17–19). In this case, both the cervix and fallopian tubes were positive for P16 and Ki-67, suggesting a possible concurrent HPV infection in the fallopian tubes. However, due to technical limitations, testing for HPV infection in the fallopian tubes was not performed to further verify this conclusion. In this case, the patient was found to have synchronous primary squamous cell carcinomas of the cervix and fallopian tube. Currently, considering that HPV infection exists in both the cervix and the fallopian tube, we speculate that the occurrence of dual cancers in this patient may be associated with HPV infection. The reasons are as follows: firstly, the persistent HPV infection undetected previously may have led to cervical carcinogenesis. Integrating the concept of the “extended Müllerian duct system”, the fallopian tubes, which also originate from the Müllerian duct, exhibit a similar response mechanism to carcinogenic stimuli(HPV infection). Inflammatory stimulation from HPV infection may thus induce squamous metaplasia in the fallopian tubes, which may further develop into cancer. This is consistent with the metaplastic mechanism mentioned earlier in squamous cell carcinoma of the fallopian tube. Secondly, HPV encodes E6 and E7 oncoproteins, which degrade and inactivate wild-type p53 and pRb tumor suppressor proteins, leading to uncontrolled cell cycle progression and genetic instability, thereby driving squamous epithelial carcinogenesis (20, 21). Related studies have proposed the “field effect” hypothesis of HPV infection to explain dual squamous cell carcinomas in the cervix and fallopian tubes, suggesting that the “field effect” of HPV infection leads to simultaneous primary squamous cell carcinomas in different sites of the female reproductive tract (19). This case is consistent with this hypothesis. Notably, IHC of the patient’s pathological specimen revealed a mutated form of p53, and TP53 is the most frequently mutated gene in human tumors and the most common mutation in various squamous cell carcinomas (13). Further research can be conducted to investigate whether HPV infection leads to p53 inactivation and abnormalities in other signaling pathways, resulting in the occurrence of MPMN, especially multiple primary squamous cell carcinomas. This could also provide new targets and directions for the selection of treatment strategies.

Early-stage fallopian tube cancer typically presents without obvious clinical symptoms or primarily nonspecific manifestations, lacks specific tumor markers, and exhibits atypical imaging features. Importantly. Compared with ovarian carcinoma, PFTC is more common in retroperitoneal lymph nodes and tiny distant metastases, and has a poorer prognosis (22, 23). Its prognostic factors correlate with staging, age, cell type, CA125 levels, postoperative residual tumor, lymphovascular invasion, location of intra-tubal lesions, and number of chemotherapy cycles, but FIGO stage and age have consistently remained significant prognostic factors (10, 11, 24). Therefore, early detection of this disease is crucial, yet preoperative diagnosis of fallopian tube carcinoma is relatively uncommon and typically requires pathologic confirmation. This patient does not present the triad of symptoms associated with tubal cancer, namely vaginal discharge, abdominal pain, or pelvic mass. Tumor markers showed no elevation, and imaging studies revealed no positive findings. Without opportunistic salpingectomy, early-stage tubal cancer would be difficult to detect. Failure to identify the lesion promptly could lead to distant metastasis in later stages, severely impacting patient’s prognosis and quality of life. This case underscores the need for heightened clinical vigilance and recognition of OS necessity. Beyond high-risk groups, patients undergoing routine laparoscopic procedures should be offered OS after thorough risk disclosure. This approach aims to reduce the incidence of ovarian cancer and lower the risks of malignancies in other sites, while enabling early detection and treatment of fallopian tube abnormalities.

## 4 Conclusion

Multiple international retrospective studies have identified endometrial carcinoma combined with ovarian carcinoma is the most common type of MPMN in the female reproductive system (25–27). Due to the rarity of primary fallopian tube squamous cell carcinoma, only a few case reports of dual primary squamous cell carcinoma of the cervix and fallopian tubes have been documented in the literature (19, 28–30). The pathogenesis remains unclear, but it is considered related to the “field effect” of HPV infection. This case also validates this hypothesis. Clinicians must remain vigilant regarding HPV infection. Beyond standard cervical cancer screening, attention must also be paid to the impact of HPV infection on other gynecological malignancies, such as tubal carcinoma. Early detection, diagnosis, and treatment are crucial to maximize therapeutic windows, improve patients’ prognosis and quality of life. Furthermore, the benefits of prophylactic bilateral salpingectomy should be better recognized, enabling clinicians to select appropriate surgical approaches rationally. Regrettably, the absence of genetic testing in this case precludes further elucidation of the pathogenesis at the genetic level. Future studies with larger sample sizes and enhanced diagnostic tools are essential to clarify the mechanisms underlying dual primary cancers and to advance therapeutic strategies.

## Data Availability

The original contributions presented in the study are included in the article/supplementary material. Further inquiries can be directed to the corresponding author.
